# Iron-restricted pair-feeding affects renal damage in rats with chronic kidney disease

**DOI:** 10.1371/journal.pone.0172157

**Published:** 2017-02-14

**Authors:** Yoshiro Naito, Aya Senchi, Hisashi Sawada, Makiko Oboshi, Tetsuo Horimatsu, Keisuke Okuno, Seiki Yasumura, Masaharu Ishihara, Tohru Masuyama

**Affiliations:** 1 Cardiovascular Division, Department of Internal Medicine, Hyogo College of Medicine, Nishinomiya, Japan; 2 Division of Coronary Heart Disease, Department of Internal Medicine, Hyogo College of Medicine, Nishinomiya, Japan; University Medical Center Utrecht, NETHERLANDS

## Abstract

**Background:**

We have previously shown that dietary iron restriction prevents the development of renal damage in a rat model of chronic kidney disease (CKD). However, iron deficiency is associated with appetite loss. In addition, calorie restriction is reported to prevent the development of end-stage renal pathology in CKD rats. Thus, the beneficial effect of iron restriction on renal damage may depend on calorie restriction. Here, we investigate the effect of pair-feeding iron restriction on renal damage in a rat model of CKD.

**Methods:**

First, to determine the amount of food intake, Sprague-Dawley (SD) rats were randomly given an *ad libitum* normal diet or an iron-restricted diet, and the food intake was measured. Second, CKD was induced by a 5/6 nephrectomy in SD rats, and CKD rats were given either a pair-feeding normal or iron-restricted diet.

**Results:**

Food intake was reduced in the iron-restricted diet group compared to the normal diet group of SD rats for 16 weeks (mean food intake; normal diet group and iron-restricted diet group: 25 and 20 g/day, respectively). Based on the initial experiments, CKD rats received either a pair-feeding normal or iron-restricted diet (20 g/day) for 16 weeks. Importantly, pair-feeding iron restriction prevented the development of proteinuria, glomerulosclerosis, and tubulointerstitial damage in CKD rats. Interestingly, pair-feeding iron restriction attenuated renal expression of nuclear mineralocorticoid receptor in CKD rats.

**Conclusions:**

Pair-feeding iron restriction affected renal damage in a rat model of CKD.

## Introduction

The prevalence of chronic kidney disease (CKD) is increasing, and this public health problem can lead to cardiovascular diseases and death. Although management of CKD reduces the risk of cardiovascular diseases and death, controversies exist regarding the benefits of some recommended treatment targets in CKD patients [1]. To reduce the morbidity and mortality in CKD patients, it is important to identify the risk factors that affect the progression of CKD.

Iron is a necessary element for maintaining the physiological homeostasis involved in cell metabolism. In contrast, excess iron can lead to free radical damage, thereby resulting in tissue damage. Importantly, iron is associated with the pathogenesis of CKD. In fact, iron deposits have been detected in proximal tubules in human CKD patients and animal models of CKD [2–5]. In this regard, we have previously reported that dietary iron restriction prevents the development of renal damage and further deterioration of preexisting renal damage in a rat model of CKD [6,7].

However, iron deficiency is associated with appetite loss [8,9]. In addition, it has been reported that calorie restriction prevents the development of end-stage renal pathology in a rat model of CKD [10,11]. Taking these findings into consideration, it is possible that the beneficial effect of iron restriction on renal damage may depend on calorie restriction.

Here, we hypothesize that the beneficial effect of iron restriction on renal damage in CKD rats is dependent upon iron restriction. In the present study, we investigate the effect of iron restriction on renal damage in CKD rats using pair-feeding experiments.

## Materials and methods

### Ethics statement

All of our experimental procedures were approved by the Animal Research Committee of Hyogo College of Medicine (protocol #13–036), and were performed in accordance with the Guidelines on Animal Experimentation that were released by the Japanese Association for Laboratory Animal Science.

### Animals

#### Protocol 1

Male Sprague-Dawley (SD) rats were purchased from Oriental Yeast Co., Ltd. (Tokyo, Japan). Six-week-old SD rats were randomly divided into two groups and were given an *ad libitum* normal chow diet (n = 6) or an iron-restricted diet (n = 6) for 16 weeks. The nutrients of a normal diet consist of 33% cornstarch, 22% casein, 5% cellulose, 30% sucrose, 5% corn oil, 4% mineral mixture, and 1% vitamin mix. Mineral mixture contains 0.43% CaHPO_4_∙2H_2_O, 34.31% KH_2_PO_4_, 25.06% NaCl, 0.623% FeC_6_H_5_O_7_∙5H_2_O, 4.8764% MgSO_4_, 0.02% ZnCl_2_, 0.121% MnSO_4_∙5H_2_O, 0.156% CuSO_4_∙5H_2_O, 0.0005% KI, 29.29% CaCO_3_, 0.0025% (NH_4_)_6_M_O7_O_24_∙4H_2_O, and 5.11% microcrystalline cellulose. An iron-restricted diet was based on a normal diet, but with a mineral mixture without adding FeC_6_H_5_O_7_∙5H_2_O. Rats were maintained on a 12hr light/dark cycle and had free access to food and water. The amount of food intake and body weight were measured every week. The amount of daily food intake was calculated as the weekly intake divided by 7.

#### Protocol 2

Six-week-old SD rats were fed on a normal diet for 1week. Afterwards, after anesthetization (ketamine HCl (50 mg/kg) and xylazine HCl (10 mg/kg), intraperitoneally), CKD was induced by cauterizing the upper and lower poles of the right kidney followed by resecting the left kidney 1 week later, as previously described [12]. After 1 week surgery, 5/6 nephrectomized rats were randomly divided into two groups and were given a normal pair-feeding diet (CKD pair-feeding [CKD-PF] n = 6) or an iron-restricted pair-feeding diet (CKD iron-restricted pair-feeding [CKD-IRPF] n = 6) for 16 weeks. Sham-operated animals given a normal pair-feeding diet were served as a control (Control pair-feeding [Control-PF] n = 6). All rats were given 20 g/day in each diet every day. After 16 weeks diet, rats were sacrificed under anesthesia with isoflurane. The adequacy of anesthesia was confirmed by disappearance of eyelid reflex, corneal reflex, loss of muscular tone, and no response to surgical manipulation. The blood was quickly withdrawn by abdominal aorta puncture and the serum and plasma were stored at -80°C before analysis. The kidney and aorta were resected and washed in phosphate-buffered saline. Afterwards, the organs were quickly snap-frozen in liquid nitrogen and stored at -80°C.

### Assessments of urine, blood, and blood pressure

24-hour urine samples were collected in metabolic cages every 4 weeks after surgery. Urinary concentrations of total protein, sodium, and potassium were determined by pyrogallol red method and electrode method, respectively (SRL, Tokyo, Japan) [7]. Peripheral blood cell count was measured using an automatic cell count analyzer (Pentra 60 LC-5000, Horiba, Kyoto, Japan). Serum iron levels were determined as previously reported [6]. Systolic blood pressure (SBP) was measured by a non-invasive computerized tail-cuff system in conscious rats every 2 weeks after surgery (MK-2000, Muromachi Kikai, Tokyo, Japan) [7].

### RNA extraction and quantitative real-time PCR

Total RNA was extracted from the kidney using TRIzol reagent (Invitrogen, Carlsbad, CA, USA). RNA was reverse-transcribed into cDNA using random primers (Applied Biosystems, Alameda, CA, USA). Real-time polymerase chain reaction (PCR) reactions were performed using the ABI PRISM 7900 with TaqMan Universal PCR Master Mix and TaqMan Gene Expression Assays (Applied Biosystems) [13]. TaqMan Gene Expression Assays used as primers and probes for each gene were as follows: *collagen type III* (assay ID Rn01437683_m1), *tumor necrosis factor-α (TNF-α)* (assay ID Rn99999017_m1), *serum and glucocorticoid-regulated kinase 1 (SGK1)* (assay ID Rn00570285_m1), and *glyceraldehyde-3-phosphate dehydrogenase (GAPDH)* (assay ID Rn99999916_s1). GAPDH was used as an internal control.

### Western blot analysis

The total protein homogenate from the aorta and the nuclear extracts from the kidney were separated by sodium dodecyl sulphate–polyacrylamide gel electrophoresis and transferred onto polyvinylidene difluoride membranes. The expression levels of proteins were detected by an enhanced chemiluminescence kit (Thermo Scientific, Rockford, IL, USA) using Image Quant LAS 4000 mini (GE Healthcare UK Ltd, Little Chalfont, UK). Here, the antibodies used were against rabbit anti-phosphorylated (Thr202/Tyr204) extracellular signal-regulated kinase (ERK), rabbit anti-ERK (Cell Signaling Technology, Danvers, MA, USA; dilution 1: 1000), rabbit anti-mineralocorticoid receptor (MR) (Perseus Proteomics, Tokyo, Japan; dilution 1: 1000), and rabbit anti-cAMP response element binding protein (CREB) (Millipore, Billerica, MA, USA; dilution 1: 1000).

### Histomorphometric analysis

The kidney and aorta tissues were fixed with buffered 4% paraformaldehyde, embedded in paraffin, and cut into 4-μm-thick sections. Periodic acid-Schiff staining was performed using serial kidney sections. Masson’s trichrome staining was performed using serial kidney and aorta sections. Glomerular lesions and tubular lesions were evaluated by semiquantitative score using the method as previously described [7]. Kidney sections were immunohistochemically stained with a primary mouse anti-desmin antibody (Dako, Glostrup, Denmark; dilution 1:50). Immunostains were visualized with the use of an avidin-biotin-peroxidase conjugate and 3,3’-diaminobenzidine substrate. Every section was counterstained with hematoxylin. Quantification of desmin positive cells was performed by counting the number of desmin positive cells in 60 glomeruli in 10 randamly selected fields.

### Electron micrography

For electron microscopy analysis, fresh kidney tissues were fixed with ice-cold buffer containing 4% paraformaldehyde, 5% glutaraldehyde, and 0.2 M Phosphate buffer (pH 7.4). Tissues were visualized on a JEM 1220 electron microscope.

### Statistical analysis

Data are presented as the means ± the standard errors of the mean (SEM). Statistical analysis was performed using Mann-Whitney U test. Kruskal-Wallis test was performed to detect differences among three groups. A value < 0.05 was considered to be significant. Statistical analyses were performed using R commander (version 1.6–3).

## Results

### Comparison of food intake and body weight between SD rats given a normal diet or an iron-restricted diet

**Fig 1:**
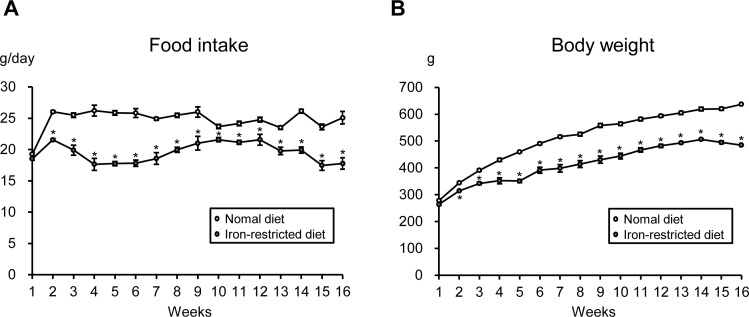
Comparison of Food Intake and Body Weight between SD Rats Given a Normal Diet or an Iron-restricted Diet. Time course of (A) the amount of food intake and (B) body weight in an *ad libitum* normal diet (white circle) and iron-restricted diet (gray circle) groups (n = 6 in each group). *p < 0.05 versus the normal diet group.

First, to determine the amount of daily food intake, six-week-old male SD rats were randomly given an *ad libitum* normal diet or an iron-restricted diet for 16 weeks, and the food intake was measured. As shown in Fig 1A, starting at 2 weeks after introduction of the diet, the amount of food intake was reduced in the iron-restricted diet group compared to the normal diet group of young, growing SD rats, and the amount of daily food intake was essentially unchanged in each group throughout the experimental period. The mean daily food intake amounts were 25 g/day in the normal diet group and 20 g/day in the iron-restricted diet group. The body weight gradually increased in both groups, but the amount of this increase was lower in the iron-restricted diet group than in the normal diet group of SD rats over 16 weeks (Fig 1B).

### Effect of iron-restricted pair-feeding on renal function and structure in CKD rats

**Fig 2:**
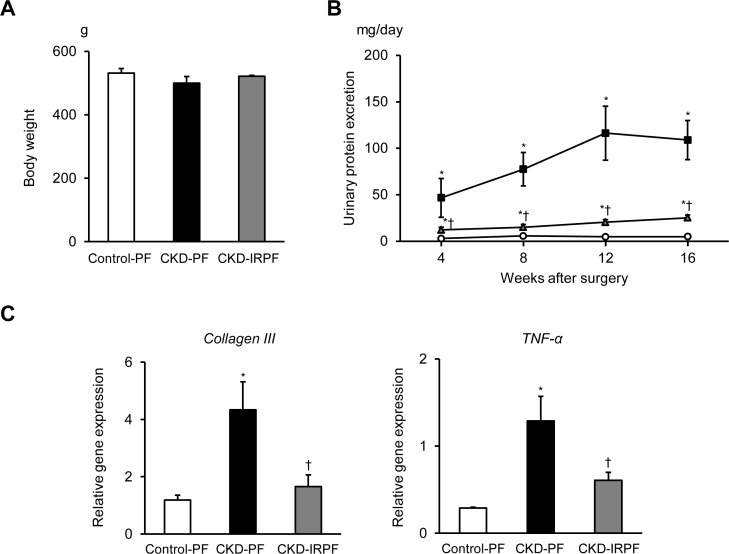
Effect of Iron-Restricted Pair-Feeding on Proteinuria and Renal Gene Expression in CKD Rats. (A) Body weight at 16 weeks after a 5/6 nephrectomy in the Control-PF (white bar), CKD-PF (black bar), and CKD-IRPF (gray bar) groups (n = 6 in each group). (B) Time course of urinary total protein excretion in the Control-PF (white circle), CKD-PF (black square), and CKD-IRPF (gray triangle) groups (n = 6 in each group). (C) Renal gene expression of *Collagen III* and *TNF-α* at 16 weeks after a 5/6 nephrectomy in the Control-PF (white bar), CKD-PF (black bar), and CKD-IRPF (gray bar) groups (n = 6 in each group). Control-PF, Sham-operated rats fed a normal pair-feeding diet; CKD-PF, 5/6 nephrectomized rats fed a normal pair-feeding diet; CKD-IRPF, 5/6 nephrectomized rats fed an iron-restricted pair-feeding diet. *p < 0.05 versus the Control-PF group, ^†^p < 0.05 versus the CKD-PF group.

Based on the initial experiments, CKD rats received either a pair-feeding normal or iron-restricted diet (20 g/day) for 16 weeks. As shown in Fig 2A, iron-restricted pair-feeding did not affect body weight in CKD rats. In contrast, blood hemoglobin, hematocrit value, and serum iron levels were decreased in the CKD-IRPF group compared to the other groups (Table 1). To examine the effects of iron-restricted pair-feeding on renal function and structure, we first assessed proteinuria in the experimental groups. A 5/6 nephrectomy induced proteinuria in both pair-feeding normal or iron-restricted diet groups compared to the Control-PF group, while proteinuria was attenuated in the CKD-IRPF group compared to the CKD-PF group (Fig 2B). Additionally, renal gene expression of *collagen type III* and *TNF-α* was increased in the CKD-PF group compared to the Control-PF group, while these increases were attenuated in the CKD-IRPF group (Fig 2C).

**Table 1:** Hematologic parameters in all groups at 16 weeks after surgery.

Parameter	Control-PF	CKD-PF	CKD-IRPF
n	6	6	6
Hemoglobin (g/dL)	12.5±0.7	11.0±0.6*	8.9±0.9*^†^
Hematocrit (%)	40.3±0.6	39.0±0.8*	35.1±2.1*^†^
Serum iron levels (μg/dL)	180.0±13.1	182.9±12.8	46.8±4.4*^†^

*p < 0.05 versus the Control-PF group.

^†^p < 0.05 versus the CKD-PF group.

Control-PF, Sham-operated rats fed a normal pair-feeding diet; CKD-PF, 5/6 nephrectomized rats fed a normal pair-feeding diet; CKD-IRPF, 5/6 nephrectomized rats fed an iron-restricted pair-feeding diet.

**Fig 3:**
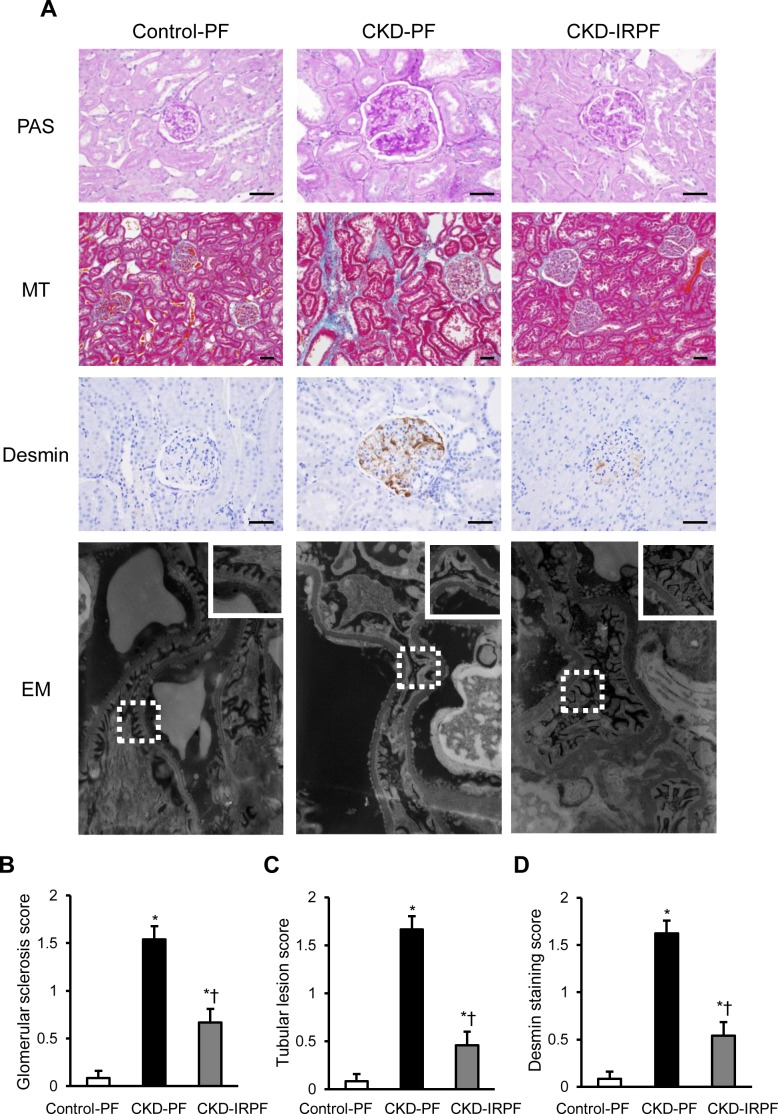
Effect of Iron-Restricted Pair-Feeding on Renal Structure in CKD Rats. (A) Representative images of periodic acid-Schiff (PAS), Masson’s trichrome (MT), desmin staining, and electron microscopy (EM) of the kidney sections. (Box area in EM is showing high magnification view.) Scale bars: 50 μm. Quantitative analysis of (B) glomerular lesion score, (C) tubular lesion score, and (D) desmin-positive cells score in the Control-PF(white bar), CKD-PF (black bar), and CKD-IRPF (gray bar) groups (n = 6 in each group). Control-PF, Sham-operated rats fed a normal pair-feeding diet; CKD-PF, 5/6 nephrectomized rats fed a normal pair-feeding diet; CKD-IRPF, 5/6 nephrectomized rats fed an iron-restricted pair-feeding diet. *p < 0.05 versus the Control-PF group, ^†^p < 0.05 versus the CKD-PF group.

We next assessed renal structure by renal histology in the experimental groups. CKD rats showed glomerulosclerosis, tubular dilatation, and cast formation at 16 weeks after a 5/6 nephrectomy compared to the Control-PF group; however, these changes were suppressed in the CKD-IRPF group compared to the CKD-PF group (Fig 3A and 3B). Likewise, interstitial fibrosis was exacerbated in the CKD-PF group compared to the Control-PF group, whereas it was attenuated in the CKD-IRPF group (Fig 3A and 3C). Moreover, renal desmin positive cells were increased in the CKD-PF group compared to the Control-PF group, while renal desmin positive cells were fewer in the CKD-IRPF group compared to the CKD-PF group (Fig 3A and 3D), suggesting that glomerular podocyte damage was less pronounced in the CKD-IRPF group compared to the CKD-PF group. Additionally, the kidneys of CKD rats showed podocyte foot processes effacement at 16 weeks after surgery compared to the Control-PF group, while these changes were reduced in the CKD-IRPF group compared to the CKD-PF group (Fig 3A). Collectively, these results demonstrate that iron-restricted pair-feeding prevents the development of proteinuria, glomerulosclerosis, tubulointerstitial damage, and podocyte injury in CKD rats.

### Effect of iron-restricted pair-feeding on hypertension and vascular remodeling in CKD rats

**Fig 4:**
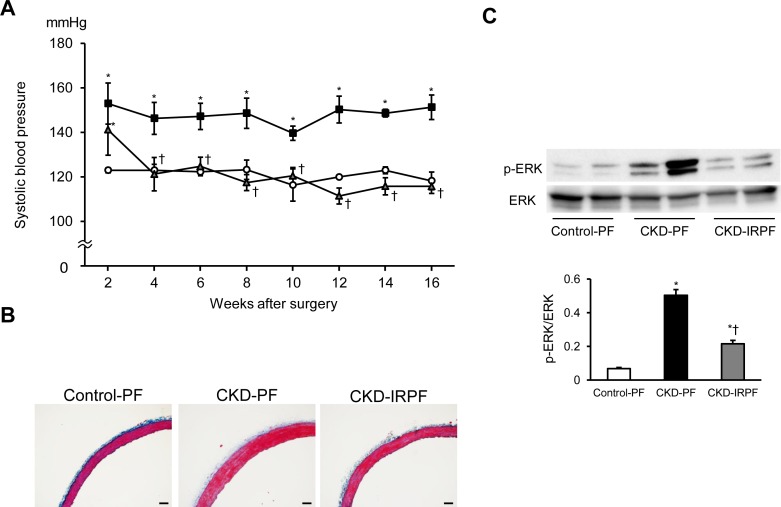
Effect of Iron-Restricted Pair-Feeding on Hypertension and Vascular Remodeling in CKD Rats. Time course of systolic blood pressure in the Control-PF (white circle), CKD-PF (black square), and CKD-IRPF (gray triangle) groups (n = 6 in each group). (B) Representative images of Masson’s trichrome staining of the aorta sections. Scale bars: 50 μm. (C) Aortic phosphorylated (top) and total (bottom) expression of ERK at 16 weeks after a 5/6 nephrectomy in the Control-PF (white bar), CKD-PF (black bar), and CKD-IRPF (gray bar) groups (n = 6 in each group). Control-PF, Sham-operated rats fed a normal pair-feeding diet; CKD-PF, 5/6 nephrectomized rats fed a normal pair-feeding diet; CKD-IRPF, 5/6 nephrectomized rats fed an iron-restricted pair-feeding diet. *p < 0.05 versus the Control-PF group, ^†^p < 0.05 versus the CKD-PF group.

CKD rats show hypertension as well as renal damage. We next assessed the effect of iron-restricted pair-feeding on hypertension and vascular remodeling in CKD rats. A 5/6 nephrectomy induced hypertension in both pair-feeding normal and iron-restricted diet groups after 2 weeks surgery (Fig 4A). SBP remained higher in the CKD-PF group than in the Control-PF group during the experimental periods, while SBP was decreased from 4 weeks after iron-restricted pair-feeding in CKD rats. Additionally, vascular hypertrophy was observed in the CKD-PF group compared to the Control-PF group, while iron-restricted pair-feeding attenuated this change after 16 weeks surgery (Fig 4B). The phosphorylation of ERK at Thr^202^/Tyr^204^ was increased in the aorta of the CKD-PF group compared to the Control-PF group, while these changes were suppressed in the CKD-IRPF group (Fig 4C). These data suggest that iron-restricted pair-feeding prevents the development of hypertension and vascular remodeling in CKD rats.

### Effect of iron-restricted pair-feeding on renal mineralocorticoid receptor signaling in CKD rats

**Fig 5:**
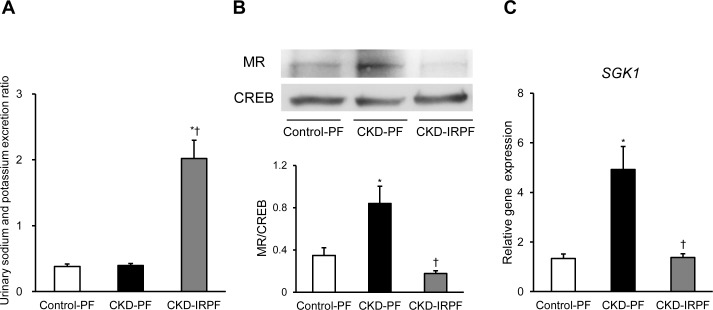
Effect of Iron-Restricted Pair-Feeding on Renal MR Signaling in CKD Rats. (A) The urinary sodium and potassium excretion ratio, (B) renal nuclear expression of MR, and (C) renal *SGK1* gene expression at 16 weeks after a 5/6 nephrectomy in the Control-PF (white bar), CKD-PF (black bar), and CKD-IRPF (gray bar) groups (n = 6 in each group). MR, mineralcorticoid receptor; CREB, cAMP response element binding protein; SGK1, serum and glucocorticoid regulated kinase 1; Control-PF, Sham-operated rats fed a normal pair-feeding diet; CKD-PF, 5/6 nephrectomized rats fed a normal pair-feeding diet; CKD-IRPF, 5/6 nephrectomized rats fed an iron-restricted pair-feeding diet. *p < 0.05 versus the Control-PF group, ^†^p < 0.05 versus the CKD-PF group.

Because we have previously reported that dietary iron restriction attenuates renal MR signaling in normal SD rats, 5/6 nephrectomized rats, and two-kidney, one-clip renovascular hypertension model rats [6,7,14], we finally assessed renal MR signaling in the experimental groups. We initially evaluated the urinary sodium and potassium excretion ratio among the experimental groups. As shown in Fig 5A, the urinary sodium and potassium excretion ratio was increased in the CKD-IRPF group compared to the other groups. Interestingly, renal MR nuclear import was increased in the CKD-PF group compared to the Control-PF group, while it was suppressed in the CKD-IRPF group (Fig 5B). Consistently, renal gene expression of SGK1, a key downstream effector of MR signaling, was increased in the CKD-PF group compared to the Control-PF group. However, it was attenuated in the CKD-IRPF group (Fig 5C).

## Discussion

To the best of our knowledge, this study provided the first evidence that iron-restricted pair-feeding attenuates the development of renal damage and hypertension in a rat model of CKD.

The prevalence of CKD is increasing, and this public health problem can lead to cardiovascular diseases and death. Although management of CKD reduces the risk of cardiovascular diseases, controversies exist regarding the benefits of some recommended treatment targets in CKD patients [1]. To reduce the morbidity and mortality in CKD patients, it is important to identify the risk factors that affect the progression of CKD. In this regard, several previous studies have shown that renal iron accumulation is increased in the tubules of CKD patients and animal models of CKD [2–5]. In addition, we have previously reported that dietary iron restriction prevents the development of renal damage and further deterioration of preexisting renal damage in CKD rats [6,7]. These observations indicate that iron may be one of the risk factors that affect the progression of CKD.

Previous studies have noted an association between iron deficiency and appetite loss [8,9]. In the present study, to investigate this association, we first assessed the amount of food intake in young, growing SD rats given an *ad libitum* normal diet or an iron-restricted diet. Beginning 2 weeks after diet introduction, the amount of food intake was lower in the iron-restricted diet group than in the normal diet group among young, growing SD rats, and the amount of daily food intake was essentially unchanged in each group throughout the experimental period. However, caloric restriction has been reported to extend longevity [15]. Regarding CKD, caloric restriction has been reported to protect against renal damage in a rat model of CKD [10,11]. In fact, compared to CKD rats fed an *ad libitum* normal diet in our previous studies [6,7], the present study found that caloric restriction (pair-feeding normal diet: 20 g/day) attenuated proteinuria in CKD rats, which is consistent with previous studies [10,11]. Although our previous report showed that dietary iron restriction prevented the development of renal damage in CKD rats [6], these observations suggest that the beneficial effect of iron restriction on renal damage may rely on calorie restriction. Therefore, in the present study, we investigated the effect of iron restriction on renal damage in CKD rats using pair-feeding experiments. Importantly, iron-restricted pair-feeding suppressed the development of renal damage in CKD rats compared to rats fed a pair-feeding normal diet.

In contrast to the beneficial effect of iron restriction on renal damage, chronic iron restriction obviously leads to iron deficiency and anemia. Indeed, in this study, iron-restricted pair-feeding induced mild iron deficiency and anemia in CKD rats compared to the other groups at 16 weeks after surgery. Therefore, in a clinical setting, iron restriction should be carefully monitored in CKD patients, and our observations may provide a balanced assessment of the risks and benefits of iron restriction in CKD patients. To prevent the development of iron deficiency and anemia, mild dietary iron restriction may be preferable for the attenuation of renal damage in CKD. Meanwhile, concerns exist regarding the correction of anemia in CKD patients. In addition, little is known about the long-term safety of iron supplementation in CKD patients. Based on our observations, it is necessary to consider total body iron stores and the role of iron in maintaining renal function in CKD patients. Additionally, correction of anemia with excessive iron supplementation should be considered in CKD patients.

A meta-analysis showed that low-calorie diets (some with low-fat components) led to a reduction of blood pressure [16]. In contrast, caloric restriction has been reported to have protective effect on renal damage in a rat model of CKD [10,11]; however, no report has shown the beneficial effect of caloric restriction on hypertension in a rat model of CKD. In the present study, iron-restricted pair-feeding attenuated the development of not only renal damage but also hypertension in CKD rats, suggesting that these beneficial effects on renal damage and hypertension are unique to iron restriction. To clarify the mechanisms of the beneficial effect of iron restriction on renal damage and hypertension, we assessed the possible mechanisms in our experimental groups. In this study, we initially found that iron-restricted pair-feeding increased the urinary sodium and potassium excretion ratio in CKD rats. Thus, we focused on renal MR signaling among the experimental groups and found that iron-restricted pair-feeding suppressed renal MR signaling in CKD rats. Interestingly, these results were consistent with our previous reports on normal SD rats, 5/6 nephrectomized rats, and two-kidney, one-clip renovascular hypertension model rats [6,7,14]. Because MR activation has been reported to be associated with the mechanism of CKD and salt-sensitive hypertension [17,18], the effect of iron restriction on MR signaling may contribute to the development of both renal damage and hypertension in CKD rats. The mechanism of the effect of iron restriction on renal MR signaling needs to be clarified in the future. In addition, a previous study reported that renal and vascular nitric oxide synthase (NOS) proteins are up-regulated in iron deficient anemic rats [19]. Therefore, renal and vascular NOS might be associated with the effect of iron restriction on renal damage and hypertension.

In this study, the experiments were performed using male SD rats. Because early studies showed that female rats absorb dietary iron more efficiently than male rats [20], it is difficult to extrapolate the results for the total population. Therefore, the effect of iron restriction might be less pronounced in female rats than in male rats.

In conclusion, we found for the first time that iron-restricted pair-feeding attenuated the development of renal damage and hypertension in a rat model of CKD. Furthermore, we showed that iron-restricted pair-feeding suppressed renal MR signaling in CKD rats.
